# 4053 A TL1 Team Approach to CNS-Localized Delivery of Glial Cell-Derived Neurotrophic Factor for Treatment of Parkinson’s Disease

**DOI:** 10.1017/cts.2020.49

**Published:** 2020-07-29

**Authors:** Shaheen Farhadi, Adithya Gopinath, Wolfgang Streit, Gregory A Hudalla, Habibeh Khoshbouei

**Affiliations:** 1 University Of Florida Clinical and Translational Science Institute; 2 University of Florida

## Abstract

OBJECTIVES/GOALS: Develop a strategy to restrict GDNF diffusion at an injected CNS tissue site for dopamine neuron rescue by endowing it with binding affinity for carbohydrates that are abundant on the cell surface and extracellular matrix. METHODS/STUDY POPULATION: GDNF will be fused to galectin-3 (G3), a human protein that binds to β-galactoside residues of cell surface and matrix glycoproteins. We characterized the binding of G3 fusion proteins to various glycoproteins and primary human myeloid cells. We incubated G3 fusions with CNS tissue ex vivo to measure their binding and depth of penetration via diffusion. We next plan to administer GDNF-G3 via CNS intracranial infusion in a murine PD model and then conduct behavioral PD phenotype testing via rotarod and pole descent to compare to non-parkinsonian controls. We will further examine the effects of GDNF-G3 on degeneration using immunohistochemical examination of post-mortem brain tissue. RESULTS/ANTICIPATED RESULTS: Based on results from previous clinical trials of GDNF delivery, we anticipate that a successful intervention using GDNF-G3 will result in rescue of midbrain dopaminergic neurons in a murine PD model. In murine CNS tissue, we observed binding to glycans at the tissue surfaces when incubated with G3 fusion proteins ex vivo, suggesting GDNF-G3 will remain localized to the injection site. Next we will administer GDNF-G3 via CNS intracranial infusion in a murine PD model and assess efficacy by behavior and histopathology. GDNF-G3-mediated dopamine neuron rescue are expected to slow or reverse the progression of PD in these animal models. DISCUSSION/SIGNIFICANCE OF IMPACT: PD treatments focus on symptomatic relief. Standard therapies have not been efficacious in rescuing of dopaminergic neurons. GDNF-G3 administered at the site of neurodegeneration would represent a milestone on the path to treating PD pathology and address limitations of GDNF delivery.

